# Foundation integrity assessment of failed buildings in Ehamufu and Aguamede, South East Nigeria

**DOI:** 10.1038/s41598-023-28043-y

**Published:** 2023-01-13

**Authors:** Kelechi Nnaji Eze, Ogbonnaya Igwe, Daniel Nnadozie Okereke, Chinenyem Stella Uwom, Kelechi Paulinus Ukor

**Affiliations:** Department of Geology, University of Nigeria, Nsukka, Nigeria

**Keywords:** Environmental sciences, Hydrology, Engineering

## Abstract

The failure of civil engineering structures especially buildings by severe cracks, partial, or complete collapse have kept the natives of communities in Aguamede and Ehamufu (Southeastern Nigeria) communities in a bothered state. Detailed geotechnical techniques and X-ray diffraction analysis were applied to investigate the soil samples from the study area. Geotechnical results revealed that the soil of the area have high clay content (62.78–82.37%), high liquid limit (48–54%) with a plasticity index of 20–28%, high moisture content (25.06–27.28%) and low permeability of 2.21 × 10^–8^–1.74 × 10^–6^ (m/sec) which hinders drainage. Maximum dry density values were in the range of 1.73–1.98(g/cm^3^) with an optimum moisture content of 17.5–19.8% and average specific gravity of 2.5 (mg/m^3^). Shear strength test revealed high cohesion (32–36.4 KN/m^2^) to low angle of intergranular friction (10–12°). Coefficient of consolidation ranges from 0.04 to 0.94 m^2^/year were observed. Coefficient of volume compressibility values were in the range of 0.00012–0.00028 m^2^/kN and showed that the soils are highly susceptible to compression as the foundations are underlain by an inadequate soil layer that is vulnerable to settlement in amount ranging from 0.553–0.654 mm/year at load pressure of 400 kN/m^2^. X-ray diffraction analysis revealed that the mineralogy of soil in the study area consist of quartz (89–89.7%) and kaolinite (10.3–11%). Statistical analyses showed that specific gravity, cohesion, clay, silt, NMC, PI, sand, LL and phi have strong interrelation in the correlation table. Comparing the geotechnical parameters from the study area with the Nigerian specification for constructions, it is shown that the study area has poor foundation materials.

## Introduction

Building Foundations are structures that transfers the weight of a civil engineering construction to the subsurface soil or bedrock^7^. Hence, foundation is a link which connects the civil construction and the supporting soil. It ought to be designed in such fashion that, the foundation soil does not collapse when subjected to stress and also settlement remains within the range of the safe limit^7,36^. Distress on buildings or eventual collapse occurs when these conditions are not met, resulting in undue settlement, development of cracks, tilt, heave or subsidence which affects the building’s serviceability. This condition is known as foundation failure, and is applicable to buildings, highway pavement, and other engineering structures^1^, Maduka and Igwe 2014). Bazant and Verdure^10^, ascribed building collapse to natural, geological or anthropogenic occurrence. The natural factors are volcanicity, subsidence, flooding and erosion, earthquakes, landslides, mud-flows, and faulting within the foundation rocks^5^, Maduka and Igwe, 2014). Geologic factors such; as rock types^36^, shrinkage and swelling clay soils^7,35^, groundwater level variation^5^. Man-made activities such as poor design and construction, hasty construction, use of low-quality materials, poor supervision^22^, unsatisfactory implementation of building codes, by the appropriate town planning officials^9,18,23^, and absence of site investigation. Subsurface geotechnical investigations as a necessity to erecting any engineering structure has not been fully enforced in Nigeria which has led to building collapse resulting to severe injuries, loss of lives and economic loss of immeasurable amount^8^. Several instances of construction failures have been published in Nigeria, Folagbe (2011) and Chinwokwu^15^, listed forty-two instances of construction failures that had occurred between 1980 and 1999, although Makinde^31^, identified fifty-four cases of building collapse between January 2000 and June 2007. In South-eastern Nigeria alone, damages worth millions of naira annually was reported by Uduji et al.^38^, and due to the increase in population/urbanization, there has been increase in construction of civil engineering activities such as buildings, roads, bridges and pipelines which starts failing after few years of construction. Foundation studies usually provide subsurface information that assist civil engineering structures, taking into consideration the safety and economy. Some civil engineering structures built in Aguamede and Ehamufu towns of Enugu state, southeastern Nigeria were observed to have developed cracks, heaved or have totally collapsed.

### Geomorphology and climate of the area

**Figure 1:**
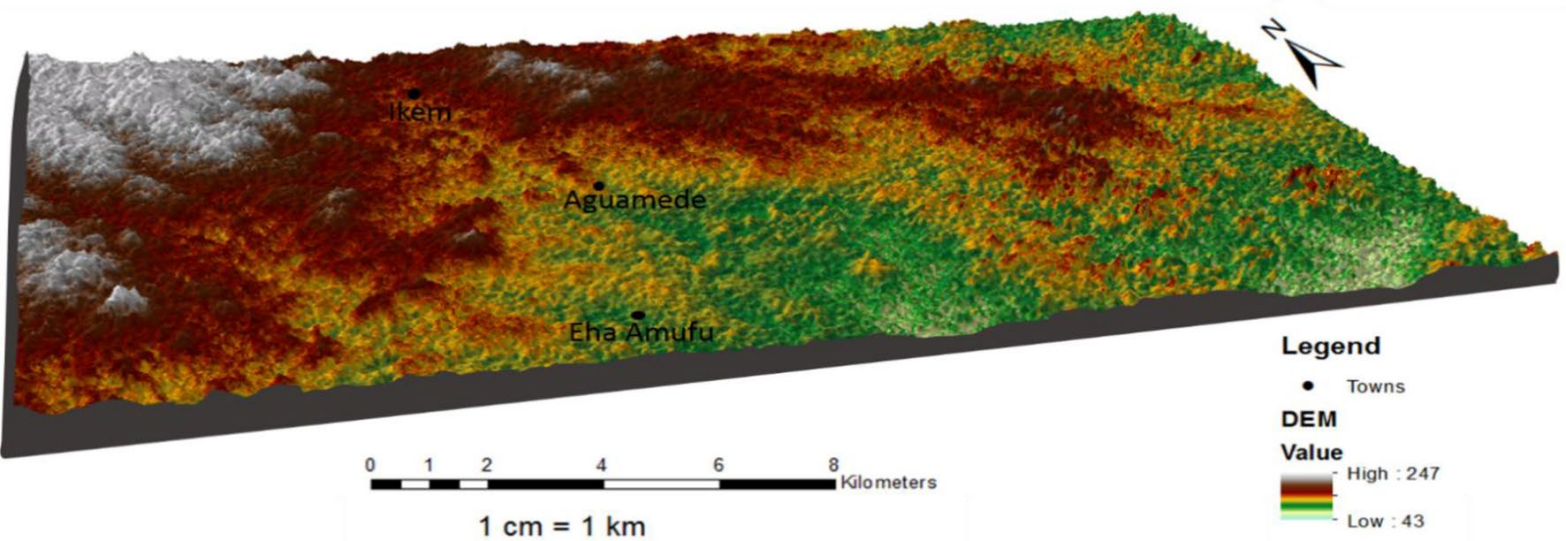
3D topographic Map of the study area.

The area of study is found in the eastern section of Enugu state and lies within latitudes of 6° 40ʹ–6° 43ʹ N and longitude of 7° 44’–7° 45 ʹ E. The area of study reveals differing elevation ranging from 260 m in the northwestern section to 80 m in the south eastern section of the area of study (Fig. 1).

**Figure 2:**
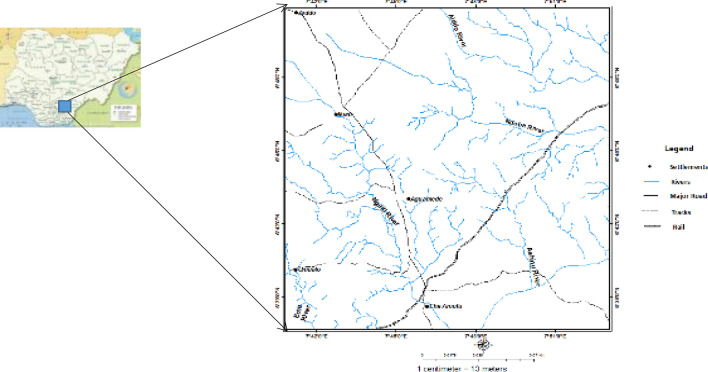
Drainage map of the study area.

The dendritic pattern of the drainage is attributed to source and flow of the rivers from the high altitude of the northern section of the area of study, flowing to the south and forms distributaries along their drainage channel (Fig. 2).

The climatic condition of the area of study belongs to a tropical sub- humid climate with two diverse seasons. The wet season lasts from May to October and the dry season from November to April. The rivers in the area of study are Nkilifi, Eme and Ashinu Rivers. Water level in hand dug wells around the drainage channels appears at a mean depth of 6 m to 15 m.

### Local geology of the area

**Figure 3:**
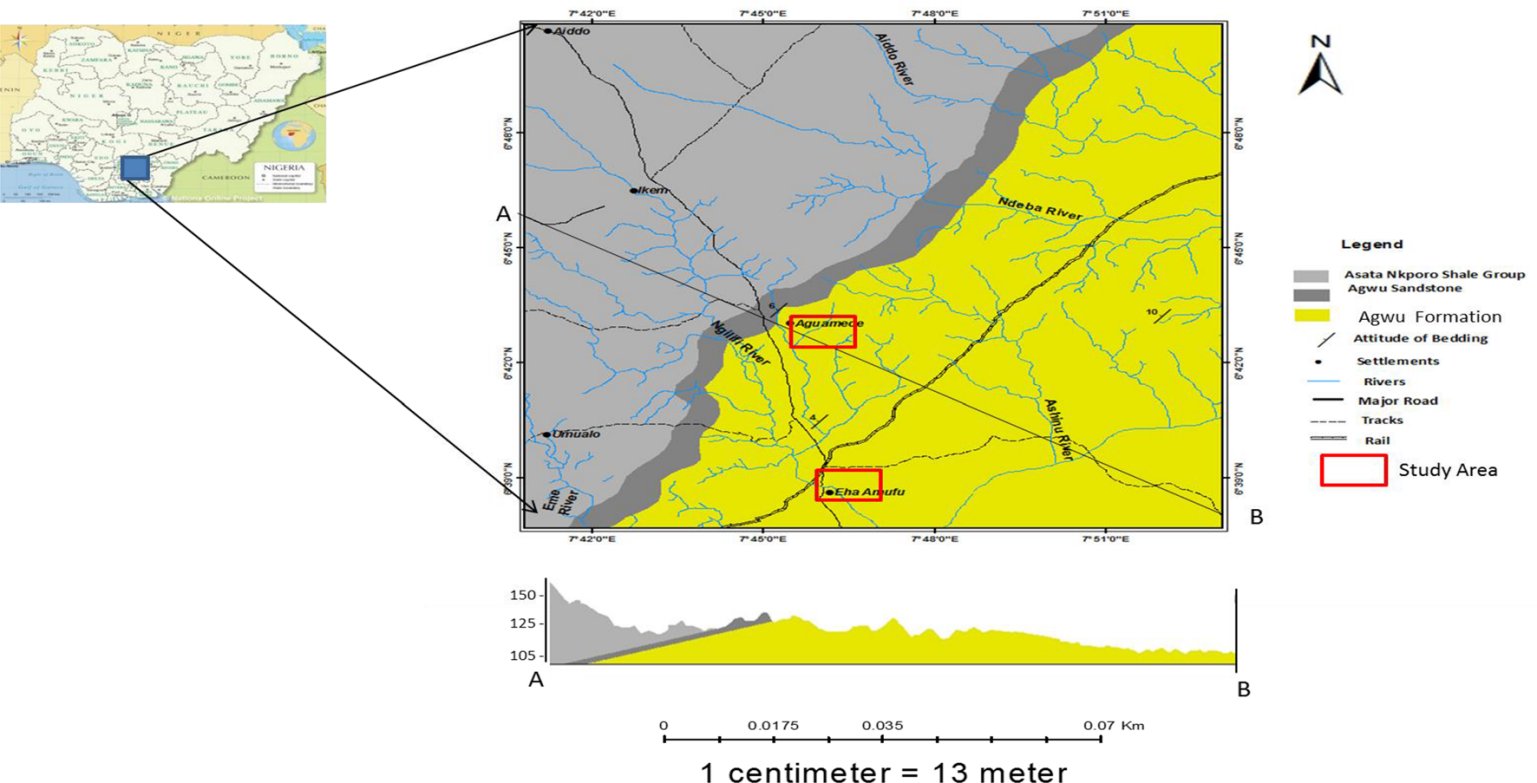
Geology map of the study area.

The study area is underlain by Agwu Formation as shown in Fig. 3 that displays the lithostratigraphic map of the study area and indicating the lithologic formation from where the samples were collected. The deposition of Agwu formation signals the end of marine sedimentation in the middle Benue Trough. It comprises of bluish grey to dark black carbonaceous shales, calcareous shales, shaley limestones, limestone, sandstone, siltstone and coal seams (Obaje, 2009). The environment of deposition of these sediments was of marine anoxic environment (the pyritic bluish grey shale), open marine with tropical conditions (the carbonates) and a shallow marine environment (the fine arenaceous facies)^4^.

## Methodology

Detailed geologic field mapping was carried out after reviewing relevant literatures and carrying out reconnaissance survey, during which the soil and rock type, topography and stream channels were identified.

### Geotechnical investigation

For the laboratory analysis, a total of ten (10) disturbed soil samples were taken from hand dug pits from Ehamufu and Aguamede, ranging between 0.5 and 1.5 m, and labeled as AG 01–AG 05, EH 01–EH 05. The samples were collected from areas with severe cracks and collapsed buildings. The various geotechnical tests performed in the laboratory includes; Sieve analysis, Compaction, Atterberg limit, Consolidation test, permeability test, natural moisture content test, specific gravity test, and triaxial test. The settlement amount was calculated based on pressure increment of 6–12 kN/m^2^, 25–50 kN/m^2^, 100–200 kN/m^2^, 200–400 kN/m^2^ with the amount of settlement to occur predicted in years. All tests were conducted in accordance with the American Society for Testing Materials (ASTM) standards and the detailed formulas of how parameters are calculated shown in Table 1 below.

**Table 1:** Showing detailed formulas of how parameters are gotten in my methodology.

Parameter	Unit	Formula	Notations and meaning
Plastic limit (L_p_)%	%	LL − PL	LL = Limit liquid
Liquidity index (LI)%	%	(W − PL)/L_p_*100	PL = Plastic limit
Consistency Index (CI)%	%	(LL − W)/Lp*100	W = Natural moisture content
Ultimate bearing capacity (Q_u_)	kN/m^2^	Q_u_ = CN_c_ + Y_w_D_f_N_q_ + 0.5Y_w_BN_y_	C = Cohesion, Y_w_ = Unit weight, D_f_ = depth of foundation(900 mm)
Safe bearing capacity (Q_s_)	kN/m^2^	Q_s_ = (Q_u_/3) + Y_w_D_f_	B = Breadth of foundation (675 mm), N_c_, N_q_, N_y_ = Bearing capacity factors, 3 = factor of safety
Coefficient of permeability (K)	m/sec	K = M_v_*C_v_*Y_w_	M_v_ = coefficient of volume compresibility, C_v_ = coefficient of consolidation, Y_w_ = Unit weight
Compresion index (C_c_)	–	C_c_ = 0.007 (LL − 10) for disturbed soil	–
Coefficient of volume compresibility (M_v_)	m^2^/kN	M_v_ = k/Y_w_C_v_	C_v_ = coefficient of consolidation, Y_w_ = Unit weight, K = coefficient of permeability
Coefficient of consolidation (C)	m^2^/year	Cv = T_v_d^2^/t	T_v_ = Time rate of settlemnt, t = time, d = drainage path
Amount of setlement	mm/year	S = C_c_/(1 + e_0_)H*Log (e_0_ + e/e_0_)	C_c_ = Compression index, e_0_ = Initial void ratio, e = void ratio, H = thickness of sample

## Results and discussion

### Geotechnical parameters

#### Grain size analysis

**Figure 4:**
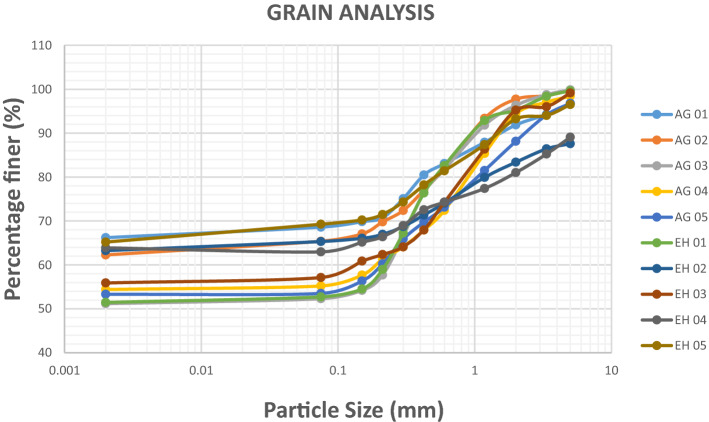
Grain size distribution curve.

**Figure 5:**
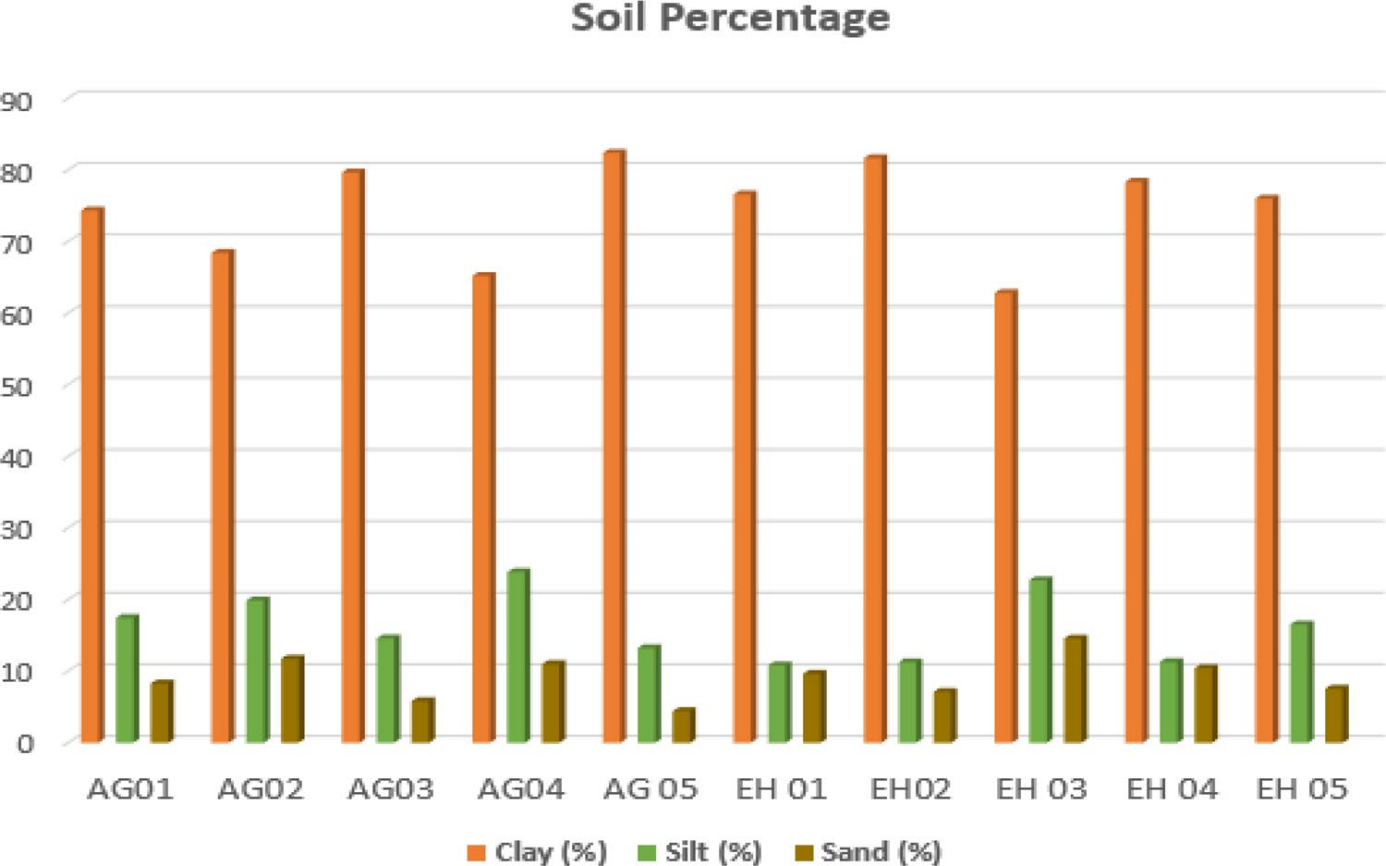
Bar chart showing the soil percentage.

The grain size distribution analysis can be utilized to determine the grading of the soil and was conducted according to ASTM D1140 (2010). The curve’s characteristics points to a somewhat fine grained soil according to Arora^7^. The result showed that the soil of the study area consists of sand within the range of 4.42–14.54%, silt ranging between 10.81 and 23.83% and clay ranging from 62.78 and 82.37% (Table 1). Figures 4 and 5 shows the percentage gradation of the analyzed soil samples and a bar charts of the soil percentages respectively. The high amount of clay percentage in the soil (62.78–82.37%.) hinders drainage within the soil which results in the saturation of clay, increase in pore pressure, decreased shear strength. The study area shows the presence of coarse materials in low percentage in the soil matrix which makes the soil vulnerable to compressibility that weakens shear strength of the soil. In accordance with the unified soil classification system (USCS), soil in the study area are categorized into three main classes; inorganic silt of high plasticity (MH), inorganic clay of high plasticity (CH) and inorganic clay of low to medium plasticity (CL), and falls within the A-7-5 and A-7-6 category using the American Association of State Highway and Transportation Official (AASHTO) classification system. Soil in this category is identified for high expansion^14^, high compressibility^6^, poor drainage and compaction characteristics, consequently making it an unsuitable foundation material.

### Natural moisture contents

The natural moisture content (NMC) test was conducted in accordance with ASTM D2216 (2010).The results of the studied area are shown in Table 2 in the range of 25.05–27.28%, thus signifying high water content. It can be seen that the studied area has similar NMC, indicating uniform geology. This NMC is suggestive of high water holding capacity of the soil, particularly at the time of this research which is at the peak of rainy season, this agrees with the particle size distribution analysis oh high clay percentage. High natural water content very much reduces the shear strength of soil, thereby causing an unceasing failure of the overlying engineering structures by inducing weathering and an increase in clay activity^30^.

**Table 2:** Summary of Geotechnical result.

Geotechnical test	Parameters	AG01	AG02	AG03	AG04	AG05	EH01	EH02	EH03	EH04	EH05
Grain size analysis	Sand %	8.27	11.71	5.81	10.99	4.42	9.62	7.12	14.54	10.41	7.53
	Silt %	17.41	19.88	14.56	23.83	13.21	10.81	11.25	22.68	11.27	16.51
	Clay%	74.32	68.41	79.63	65.18	82.37	76.57	81.63	62.78	78.32	75.96
Atterbeg limits	Liquid limit %	52	54	51	52	53	48	46	50	49	52
	Plastic limit %	24	28	31	26	27	24	25	25	27	27
	Plasticity index %	28	26	20	26	26	24	21	25	22	25
Consistency indices	Liquidity index %	9.79	− 10.81	− 19.40	1.81	− 0.73	4.42	10.86	5.84	− 6.23	− 0.52
	Consistency index%	90.21	110.81	119.40	98.19	100.73	95.58	89.14	94.16	106.23	100.52
Compaction	Max. dry density (g/cm^3^)	1.73	1.76	1.68	1.79	1.86	1.93	1.95	1.89	1.98	1.91
	Opt. moisture content (%)	19.8	18.3	19.0	18.0	19.5	17.9	18.6	17.5	16.9	17.5
Specific gravity	(G/CM^2^)	2.34	2.49	2.35	2.42	2.47	2.59	2.61	2.54	2.66	2.51
Moisture content	(%)	26.74	25.19	27.12	26.47	26.81	25.06	27.28	26.46	25.63	26.87
Permeability (K)	(m/sec)	3.15 × 10^−7^	4.27 × 10^−8^	9.03 × 10^−8^	1.74 × 10^−6^	1.56 × 10^−7^	5.24 × 10^−7^	2.21 × 10^−8^	2.24 10^−8^	6.28 × 10^−7^	3.49 × 10^−6^
Undrained triaxial Test	Cohesion (C) (KN/m^2^)	34.1	36.4	35.3	32.8	33.6	35.0	34.8	34.2	32.0	35.8
	Intergranular friction angle (Φ) (^o^)	11	9.6	10.8	11	10	15	12	14	13	12
	Ultimate bearing capacity (Qu)	347.25	353.10	359.78	360.45	329.18	379.20	356.34	330.40	328.79	388.73
	Safe Bearing capacity (Qs)	132.08	135.57	136.95	137.33	127.44	143.05	136.13	126.41	125.94	146.87
Consolidation	Compression Index (C_c_)	0.29	0.31	0.29	0.29	0.30	0.27	0.27	0.28	0.27	0.29
	Coefficient of Consolidation (C_v_) (C_v_) (M^2^/yr)	0.12	0.21	0.17	0.04	0.94	0.22	0.11	0.23	0.16	0.18
	Coefficient of Volume Change (M^2^/kN)	0.00017	0.00023	0.00013	0.00012	0.00015	0.00028	0.00016	0.00025	0.00013	0.00021
	Settlement Amount (mm/yr) at pressure load of 400kN/m^2^	0.605	0.654	0.581	0.613	0.553	0.630	0.606	0.640	0.578	0.582

### Atterberg limit

**Figure 6:**
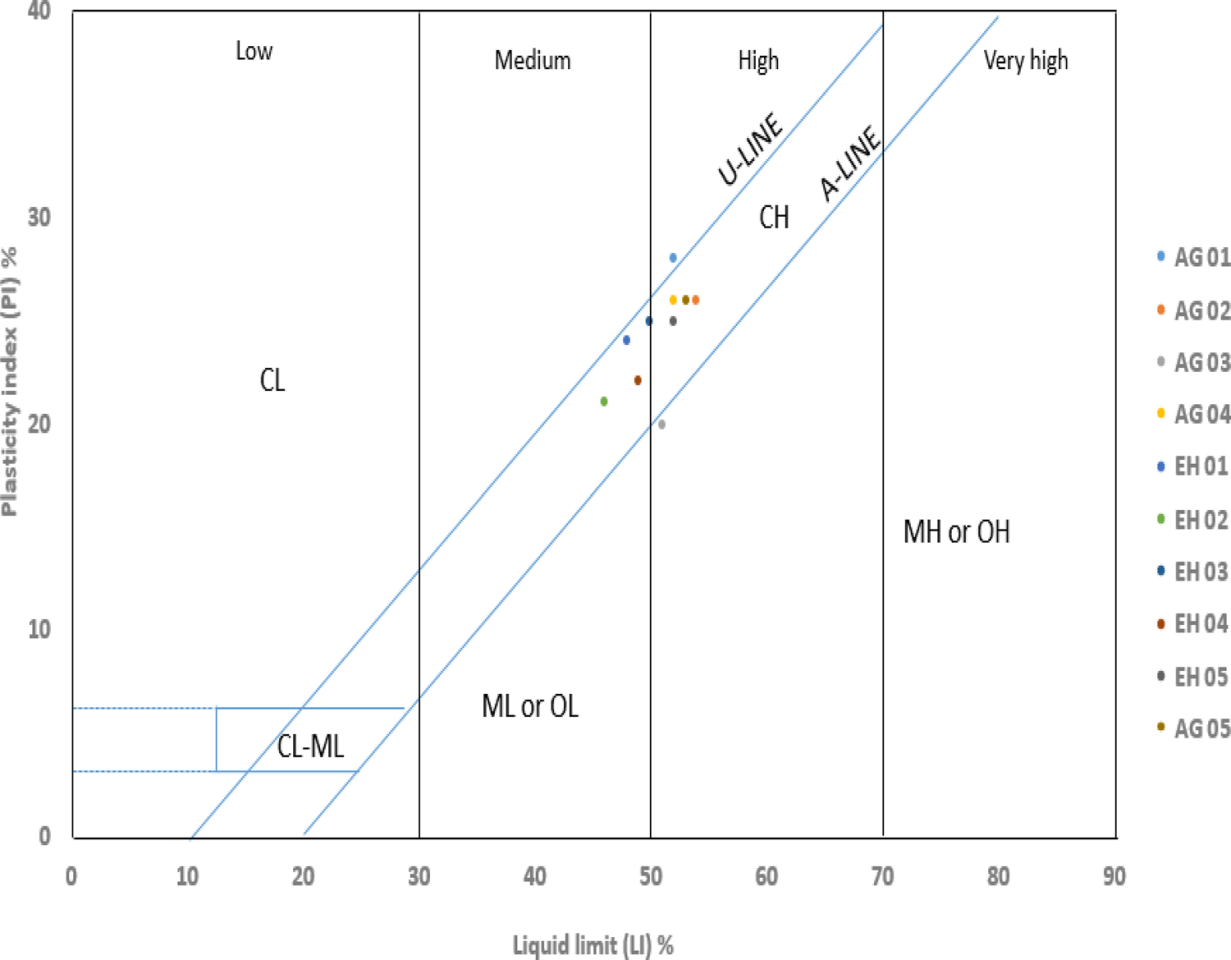
Plasticity chart of the samples^12^.

The atterbeg limit test was done in accordance with ASTM D4318 (2010) outcome of the atterbeg limits test summarized in Table 2, revealed that Liquid Limit (LL) are in the range of 46–54% and as stated by Bell^11^ (Table 3), the values of liquid limit in the area of study, displays moderate to high plasticity. The Plastic Limit (PL) ranges from 24 to 31%, while the Plasticity Index (PI) ranges from 20 to 28% and they plot above the A-line of the Casangrande plasticity chart (Fig. 6), except sample AG03 which plot below the A-line. Therefore the soils can be categorized as CL, MH or CH according to the Unified Soil Classification System (USCS) (Fig. 6). Igwe et al.^26^, recorded that soils with high clay content impede water drainage, and resulting increase in pore water pressure in the soil. In reference to the values from the result, the soils are regarded as soils with high expansive capability^14^.

**Table 3:** Soil plasticity projection using the range of liquid limits^11^.

Description		Range of liquid limit
Lean or silly	Low plasticity	< 35
Intermediate	Intermediate plasticity	35–50
Fat	High plasticity	50–70
Very fat	Very high `plasticity	70–90
Extra fat	Extra high plasticity	> 90

**Liquidity index** index range from − 19.40 to 10.86% (Table 2).The negative values are indicative that the soil is drier than the plastic limit^7^. The soil in the studied area will behave more like plastic since the values are closer to zero while sample AG01, AG03, AG05, EH04 and EH0 will behave in a brittle manner or crumbles into piece since the liquidity index are negatives.

**The Consistency Index (CI)** of the soil in the studied area ranges from 89 to 193%. Samples AG02, AG04, EH01, EH02 and EH03 are in a semi-solid state, while AG01, AG03, AG05, EH04 and EH05 which shows C.I greater than 100% are relatively firm. The values of the consistency and liquidity index suggests that the collected soil samples are relatively consistent and firm, revealing that the soil is vulnerable to alterations in consistency at varying moisture content.

### Specific gravity

The specific gravity test was performed in accordance with ASTM D854 (2010). The results shown in Table 2, ranges from 2.34 to 2.61 with a mean value of 2.75**.** Sample EH04 shows the highest specific gravity (2.83 g/cm^3^), with sample AG03 having the least specific gravity (2.65 g/cm^3^). According to Reidenouer^33^, soils with specific gravity of 2.65 and below are generally unstable and unreliable and could collapse at the engineering sites, especially with water. Roy and Dass^34^ found out that rise in specific gravity causes an increase in the shear strength parameters (intergranular friction angle and cohesion).

### Compaction

**Figure 7:**
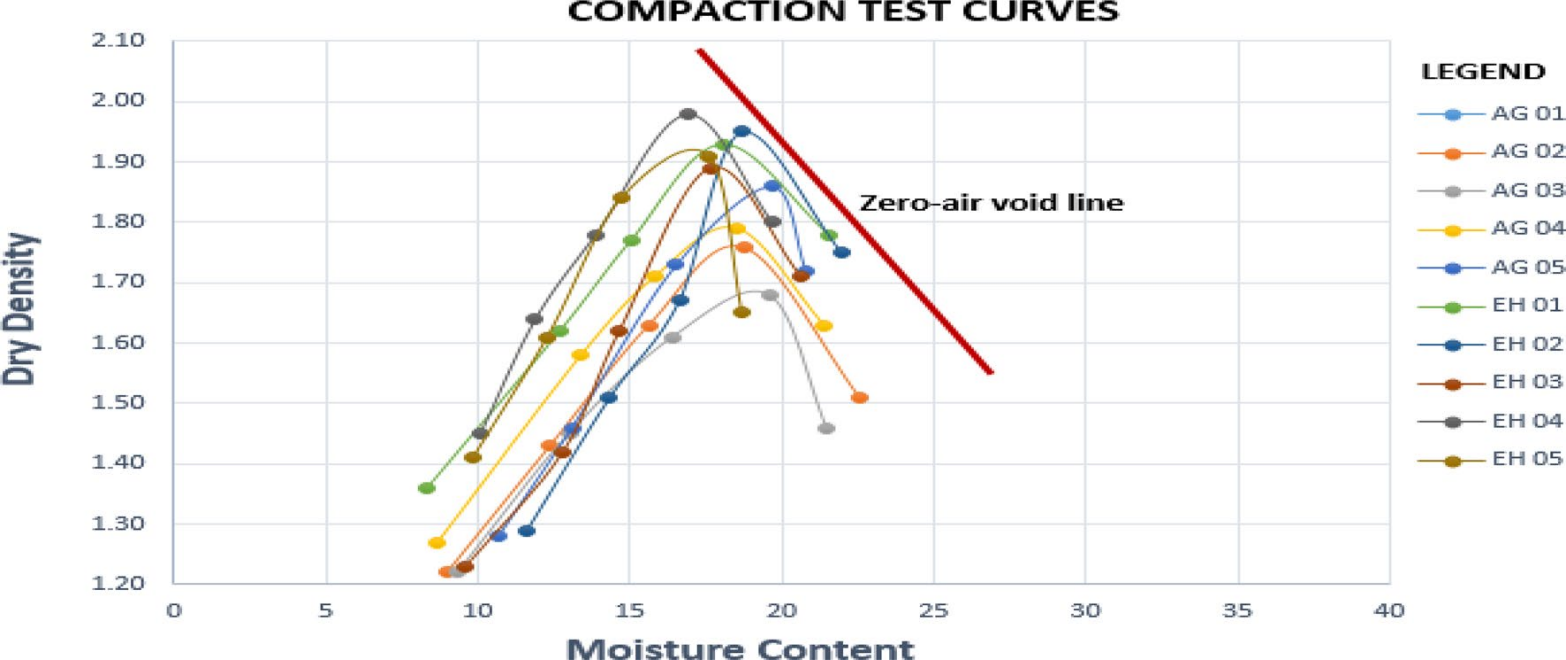
Samples compaction curves.

The compaction test was conducted following ASTM D1557 (2007). As reported by^27^, compaction tests mainly disclose how compacted or loose a soil material is, and the link connecting optimum moisture content and compaction effect. The result from the compaction test (Table 2) and the stacked compaction curves (Fig. 7) showed that the values of maximum dry densities (MDD) ranges from 1.68 g/cm^3^ to 1.98 g/cm^3^ and optimum moisture content (OMC) the ranges from 16.9–19.8%. The compaction result disclosed high optimum moisture content and poor to fair dry density (Table 4). According to Jegede^28^, the preferred materials for engineering foundation are the soils possessing high MDD at low OMC. Sample AG01, AG02 and AG03 have the lower MDD (1.73, 1.76 and 1.68) and higher OMC (19.8, 18.3 and 19.0) in the studied area respectively, which suggests an unstable soil^20^.A comparison of OMC and the natural moisture contents shows that they exist with natural moisture content higher than the OMC in its natural state. This implies that the continuous infiltration of water into the soil, will lead to resulting increase in pore pressure and reduction in shear strength leading to instability of foundation and then failure.

**Table 4:** Soil compaction Classification (Emesiobi, 2000).

MDD(g/m^3^)	General value as a sub-grade foundation material
> 2.1	Excellent
1.9–2.1	Good
1.7–1.9	Fair
1.6–1.7	Poor
1.1–1.6	Very poor

### Coefficient of permeability

The coefficient of permeability test was conducted according to ASTM 5084 (2003).The calculated coefficient of permeability of the studied area ranges from 2.21 × 10^−8^ to 3.49 × 10^−6^ m/s (Table 2). According to Casagrande and Fadum (1940), coefficient of permeability that ranges from 10^−6^ to 10^−4^ m/sec is characterized as good, 10^−8^ to 10^−6^ m/sec as poor, and 10^−11^ to 10^−8^ m/sec as impervious. The results showed lower K-values (poor), for all the soil samples and areas with expansive soils have been established to have low permeability^7,13^. The low permeability is as a result of low void ratio, poor interconnectivity, resulting from high fine content. Sowers and Sowers^37^, recorded that high plasticity denotes poor permeability and low hydraulic conductivity,suggesting that due to low drainage, clay is susceptible to water-log during rainy seasons^30^, and^39^. Furthermore, this suggests that there will be pore pressure buildup, thereby reducing the effective stress and thus, reduces the strength of foundation material when subjected to loading and cause problems.

### Undrained triaxial test

**Figure 8:**
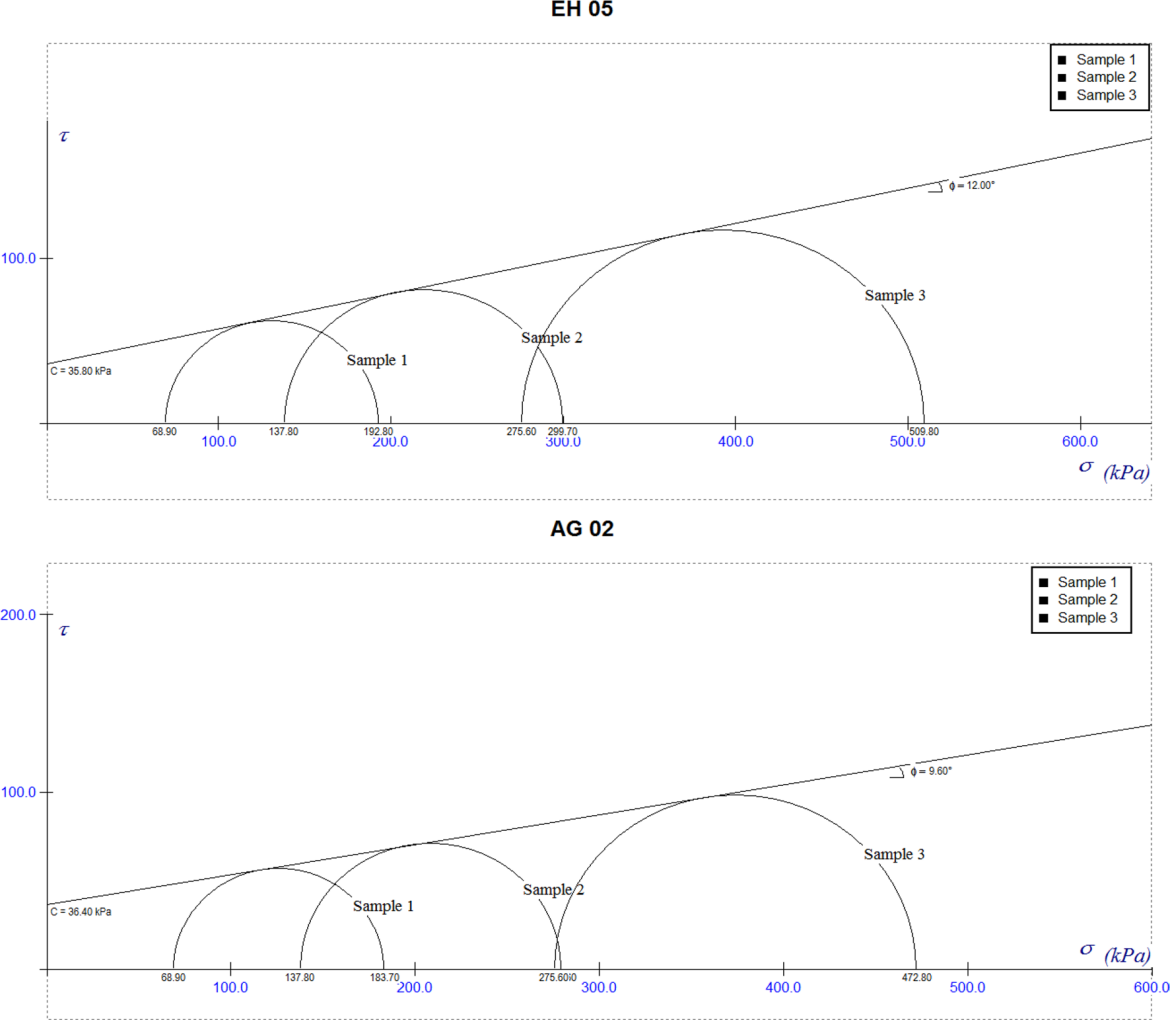
Morh view plot for EH05 and AG02.

Unconsolidated undrained test was carried out according to ASTM 2850 (2007). The shear strength parameters were obtained with the help of Mohr view application, Fig. 8, illustrates some of the slopes and their failure envelopes. The cohesion values are within the range of 32 to 35.8 kN/m^2^ while the angle of internal friction (Φ) was within the range of 9.6 to 15° (Table 2), indicating a relatively high cohesive strength to low angle of internal friction respectively. The area appears to have similar shear strength parameters and a possible uniform behavior under shear. The low frictional contact in the soil of the study area is ascribed to low sand percentage^17,21,27^. Additionally, reduction in the intergranular friction angle causes a resultant decrease in the shear strength of the foundation soil, also extreme water content reduces intergranular friction angle, (Al-shaye, 2011). Reduced intergranular friction angle and increased cohesion indicates a low shear strength, loss in bearing capacity, thereby making a site unsuitable for construction activity^39^.

The ultimate bearing capacity and the safe bearing capacity of the studied area was calculated using the Terzaghi’s equation. The bearing capacity enables geotechnical engineers in choosing the type of foundation and amount of load to place on the foundation. The calculated safe bearing capacity of the studied area ranges from 125.6 to 146.8 KN/m^2^ (Table 2), which reveals the studied area has relatively good safe bearing capacity. The high plasticity index of the area due to the high cohesive values translates to high water retention capacity, which increases the pore water pressure of the soil. Igwe and Fukuoka^25^, reported that an increase in excess pore pressure reduces the soils shear strength. A rise in pore pressure decreases the effectiveness of the soil which in turn affects the safe bearing capacity and leads to cracks on the walls, differential settlements and sinking. This failure is mostly due to the incompetence of clay as a swelling and shrinking materials.

### Consolidation test

**Figure 9:**
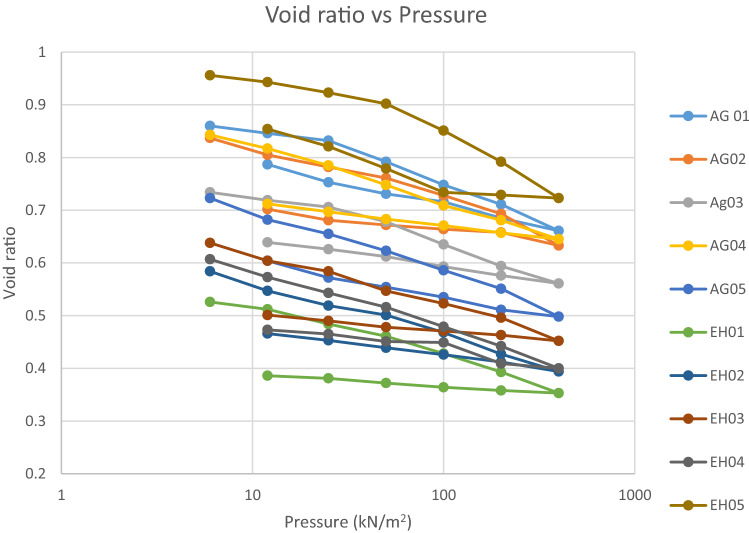
Consolidation curve for all the studied sample.

The consolidation test was carried out in accordance with ASTM D2435. Coefficient of consolidation could be utilized to establish the settlement rate of engineering structures constructed on a compressible foundation material like clay, whereas the coefficient volume compressibility is used to evaluate the settlement amount of the engineering construction. The plot of pressure versus void ratio in Fig. 9, was done so as to calculate rate of settlement and amount of settlement.

**Figure 10:**
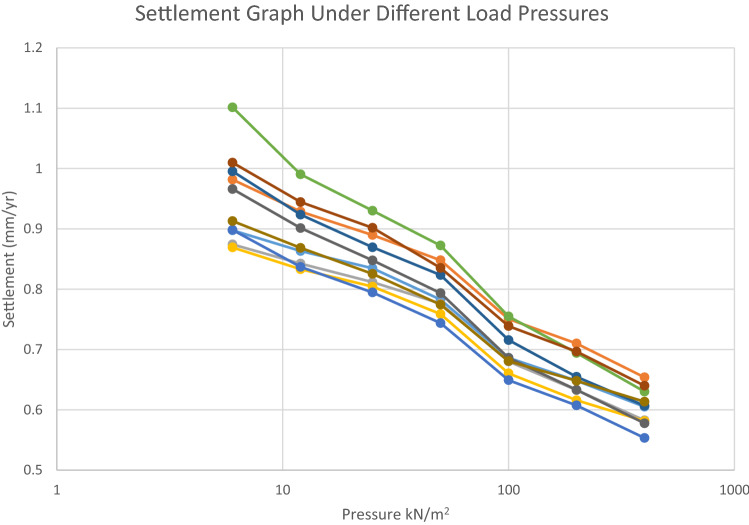
Graph showing settlement under different load pressures.

The values of Coefficient of consolidation shows the compression rate of the studied soil. The ranges of coefficient of Consolidation (Cv) values of the area are from 0.04 to 0.94 m^2^/year were observed. Coefficient of volume compressibility values were in the range of 0.00012 to 0.00028 m^2^/kN showing that soils in the locations are highly susceptible to compression due to the fact the foundations are underlain by an unsuitable soil which is vulnerable to settlement in amount ranging from 0.553 to 0.654 mm/year at a pressure load of 400 kN/m^2^ as seen in Table 5 showing the amount of settlement for different pressure loads, further explained by the settlement graph (Fig. 10). Head and Epps^24^ suggested that soils with coefficient of volume compressibility value between 0.0001 to 0.0003 m^2^/kN (0.1–0.3 mPa^−1^) have medium compressibility while soils with coefficient of volume compressibility values between 0.00031 to 0.0015 m^2^/kN (0.31–1.5 mPa^−1^) have high compressibility. This implies that the soil samples from the study area have medium compressibility. Ale (2012) implied that settlement amount of 1 mm/year is the prescribed requirement of failure below which the settlement is low and above which the settlement amount is good. As the load pressure increases, the soil void ratio decreases and due to low permeability of the soil translated by the low values of the Mv and Cv, high cohesive strength, which agrees with the result of sieve analysis. When a low permeability soil is subjected to structural loading, the water that saturates the pore spaces of the soil carries it initially leading to an accelerated rise in pore water pressure. The stress is transferred to the soil skeleton when the excess pore water pressure is being discharged as water is drained away from the void spaces in the soil, resulting in the gradually compression of the soil coupled with the shrinking of clay soil during dry season and dispersion of kaolin in the presence of water during wet season leading to undue settlements evidenced by cracks, collapse of part or all of the structure (Fig. 9). Consolidation in clay soil may last for many years or even decades unlike sandy soil where consolidation is rapid.

**Table 5:** Amount of settlement at different pressure loads.

Pressure (kN/m^2^	AG 01	AG 02	AG 03	AG 04	AG 05	EH 01	EH 02	EH 03	EH 04	EH 05
6	0.897563	0.981609	0.874152	0.869111	0.898168	1.1013297	0.995275	1.009769	0.965943	0.912881
12	0.863127	0.928571	0.842319	0.832954	0.836869	0.990289	0.923436	0.944346	0.901257	0.868385
25	0.834502	0.889672	0.811623	0.8042	0.794497	0.930078	0.869349	0.901658	0.847651	0.825347
50	0.78259	0.847902	0.776051	0.758641	0.743704	0.872359	0.823379	0.835215	0.793391	0.774385
100	0.686106	0.750497	0.680116	0.660396	0.649061	0.754763	0.715507	0.738736	0.685998	0.680574
200	0.648213	0.709875	0.632666	0.61583	0.607279	0.694161	0.654671	0.696524	0.632941	0.647961
400	0.605019	0.653751	0.582479	0.581228	0.55322	0.630115	0.606726	0.639861	0.577566	0.61337

**Figure 11:**
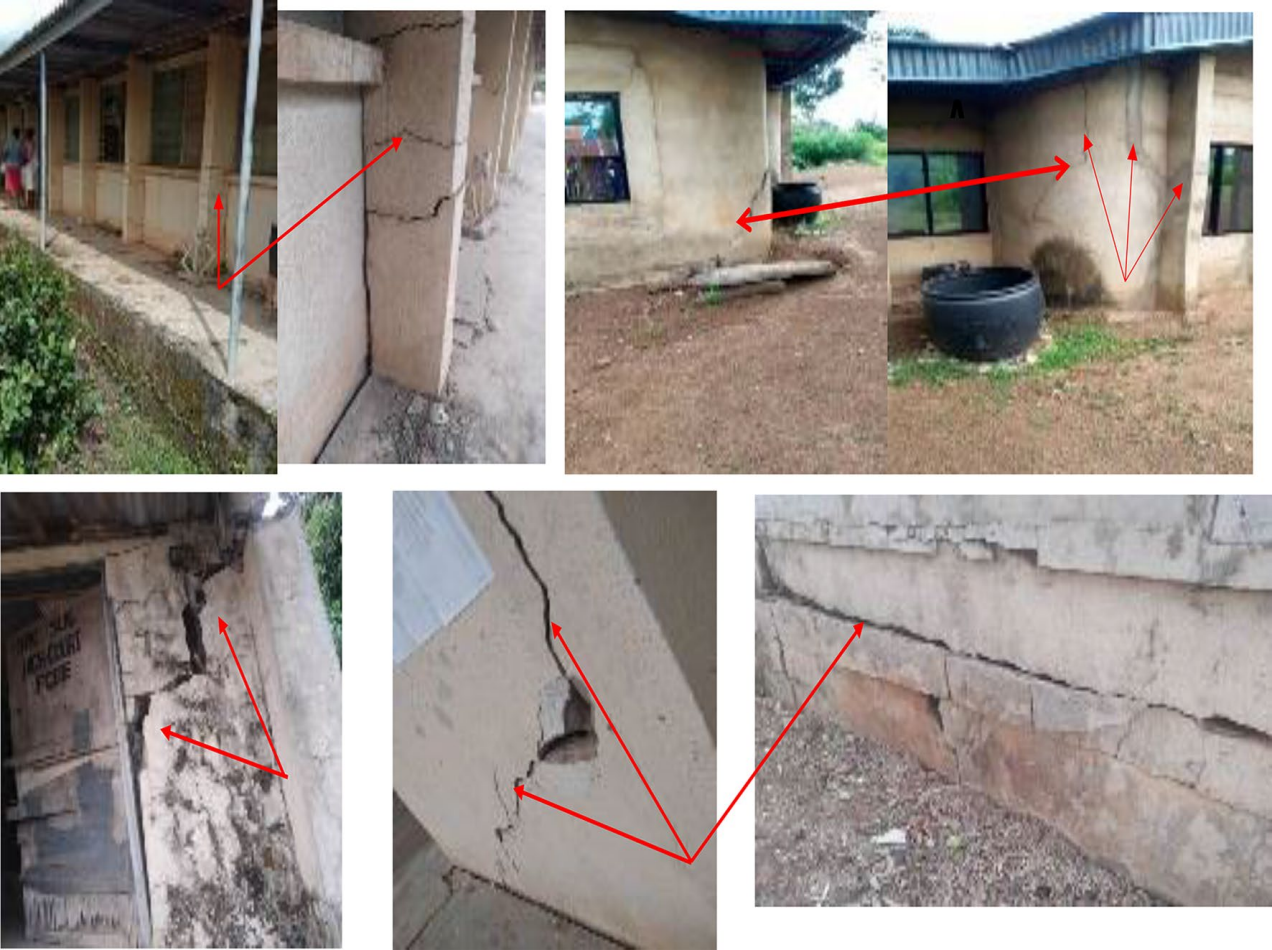
Horizontal, Diagonal and vertical cracks on buildi.

### X-ray diffraction analysis

**Figure 12:**
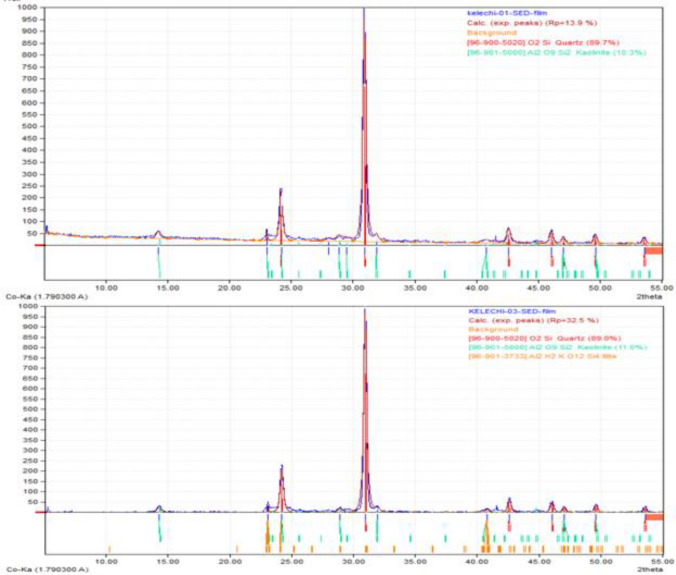
XRD displaying the mineral constituents of the soil samples.

X-ray diffraction analyses was performed on the samples to distinguish the minerals present in the soil and the result of the test is shown in Fig. 12. The minerals present in soil samples are quartz (89.0–89.7%) and kaolinite (10.3–11.0%). Kaolinites are the most prevalent clay mineral. They display low cation exchange and are principally formed by hydrothermal alteration or weathering of feldspars and usually occurs with quartz and oxides, siderite and muscovite^40^.llite and hydro muscovite are produced as a result of weathering of muscovite and disintegrates futher to form montmorillnite and finally kaolinite with increase in water. Kaolinite clays belongs to the Kandite group of minerals. At high water content, kaolinite clay has very limited inherent strength and will readily disperse^32^ leading to failure. The presence of clay minerals has the tendency of increasing the soil’s plasticity, which is an important cause of foundation instability and failure.

### Multivariate statistical analysis of the foundation soil

#### Pearson’s correlation analysis

The Pearson’s correlation analysis is shown in Table 6. The LL, NMC, OMC and clay are seen to connect negatively (− 0.6381, − 0.60511, − 0.6981 and − 0.57997) with specific gravity, while MDD, Phi and silt correlate positively (0.929825, 0.606876 and 0.663836) with specific gravity. The negative correlation translate to a decrease in specific gravity as the NMC, LL and clay increases, this confirms the low specific gravity of the soil because of high water retention. Furthermore an increase in MDD, Phi and silt increase the soil specific gravity. The consequences of water content and clay on building structures are related^16^, the effects of high clay content which signifies water affinity may be answerable for the poor engineering properties, which causes cracks and foundation instability in engineering structures. The noted negative correlations means an inverse proportionality between the parameters; thus an increase in the percentage of clay increases the soil’s cohesive strength. Also clay was observed to have a strong positive correlation with cohesion and NMC (0.813 and 0.691 respectively) and a strong negative correlation with silt and sand (**− **0.971 and − 0.954), this can be credited to their solid connection with soil plasticity^29^. Soil cohesion is recognized to increase with increase in clay content and reduces if it is silt or if the soil contains more of sand, this conforms to the sieve analysis and atterberg limits results. This positive association translate to an increase in MDD and Phi, with an increase in silt percentage.

**Table 6:** Pearson correlation matrix of the geotechnical parameters.

Parameters	G (mg/m^3^)	LL (%)	PI (%)	NMC (%)	OMC (%)	MDD (g/cm^3^)	Cohesion	Phi	Mv (M2/KN)	Cv (M2/year)	clay%	silt %	sand
G (mg/m^3^)	1												
LL (%)	**− 0.6381**	1											
PI (%)	**− **0.35735	**0.640336**	1										
CI (%)	**− **0.23607	0.409462	**− **0.38933										
NMC (%)	**− 0.60511**	**− **0.05385	**− **0.1899	1									
OMC (%)	**− 0.6981**	0.297715	0.249607	0.444062	1								
MDD (g/cm^3^)	**0.929825**	**− 0.62939**	**− **0.22904	**− **0.19793	**− 0.62553**	1							
Cohesion	**− **0.18096	0.171323	**− **0.02593	**− **0.02829	0.175621	**− **0.28838	1						
Phi	**0.606876**	**− 0.69881**	**− **0.24061	**− **0.31779	**− 0.59752**	**0.64671**	**− **0.16089	1					
Mv (M2/KN)	**− **0.14578	**− **0.04287	**− **0.27581	**− **0.18007	**− **0.00943	**− **0.27321	0.489578	0.291613	1				
Cv (M2/year)	**− **0.02026	0.32273	0.211643	0.090031	0.381665	0.081563	**− **0.09136	**− **0.2756	**− **0.10512	1			
Clay %	**− 0.57997**	**0.549861**	**0.838142**	**0.6914**	0.306789	**− **0.47051	**0.8128**	**− **0.19479	**− **0.11469	**− **0.111111	1		
Silt %	**0.663836**	**− 0.52543**	**− 0.77286**	0.131344	**− **0.44229	**0.596862**	**− **0.01687	0.304516	0.119621	0.14775815	**− 0.971037**	1	
Sand	0.411742	**− **0.39642	**− 0.83284**	0.137078	**− **0.22057	0.249513	**− **0.03489	0.077076	0.196189	0.14105756	**− 0.954247**	**0.894228**	1

Significant values are in bold.

### Correlating the engineering qualities of the study area with Nigerian Specification

The geotechnical properties of the studied area was compared with the Nigerian requirement for construction (adapted from^2^ (Table 7). The soil in majority of the locations belong to the CH and A-7–6 class of the USCS and AASHTO classification accordingly. Thus suggesting that they probably have comparable geotechnical characteristic. Sowers and Sowers^37^, Aghamelu et al*.*^3^, and Maduka et al.^30^, disclosed that CH soils are linked with low to medium compaction, high compressibility, high expansive properties, poor drainage and low shear strength with change in moisture contents. Taking into account the range of the LL, PI and percentage of clays, the soils are highly compressible.

**Table 7:** Comparing the results of the study area, with the Nigerian requirement for construction.

Properties of materials	Nigerian specification	Study area
General filling and embankment		
MDD	> 0.047	1.68–1.98
OMC (%)	< *18*	16.9–19.8
LL (%)	< *40*	48–54
PI (%)	< *20*	20–28
Passing no #200	≤ *35*	77–95
Soaked CBR (BS)	> *5*	Not tested
Sub-base course		
LL (%)	< 35	48–54
PI (%)	< *16*	20–28
CBR West African standard	≥ *25*	Not tested
Base course		
LL (%)	≤ *30*	48–54
PI (%)	≤ *13*	20–28

Significant values are in italics.

Comparing the geotechnical parameters from the area of study with the Nigerian requirement for constructions, it is observed the Atterberg limit of the study area showed slightly high LL and PI, which indicates the soil is not suitable for general fillings and embankment and as a sub grade course. The fines percentage (clay) (passing sieve no 200) is way above the standard for Nigerian specification thereby branding the soil a poor foundation material. High percentage of fines in a soil highly impacts its engineering qualities by retaining more water there by reducing the soils effective stress. The OMC showed slight variation when compared with the Nigerian specification. Thus, the area of study possess substandard foundation materials and so should be properly stabilized prior to construction activities on the soil.

## Conclusion

A combination of field exploration, geotechnical and statistical analysis were used in this research to be able to determine how the subsurface geotechnical parameters affects the foundations within the study area and to also ascertain the safe and ultimate bearing capacity so as to determine the foundation instability. The field investigation revealed noticeable cracks on the buildings probably due to weakness of the foundation, low shear strength of the foundation soil and differential settlements. The geotechnical experiments indicate that the foundation soil are made up of 4.42–14.54% sand, 10.81–23.83% silt and 62.78–82.37% clay confirming the predominance of clays, which exceed the 35% maximum set by Nigerian requirement for construction. The subsurface soil are classified into MH, CH and CL of the USCS and falls under the A-7-6 class of AASHTO classification system. This soil is known for high expansion^14^, high compressibility^6^, low compaction and drainage properties, thus making it a substandard foundation material. The soil exhibits high plasticity, poor permeability and high moisture holding capacity, this is due to the characteristics of clay being porous but least permeable, which makes the soil a substandard foundation materials. These soils exhibit high compressibility, high OMC and low to moderate MDD and an average specific gravity of 2.74 g/cm^3^, confirming high clay content of the foundation soil, which are characteristics of a poor foundation materials. The shear strength parameters disclosed that the soils possess high cohesive strength to low angle of internal friction capable of failure. The area has relatively good safe bearing capacity, but the high plasticity index due to the high cohesive values translates to high water retention capacity, which increases the pore water pressure of the soil and reduces its effectiveness thereby affecting the safe bearing capacity and leading to differential settlements, cracks on the walls and instability. Coefficient of consolidation showed that the soils are highly susceptible to compression as the foundations are carried by a substandard soil layer that is vulnerable to settlement in amount ranging from 9.65 × 10^−4^ to 1.101 × 10^−3^ m/year. Statistical analyses showed that specific gravity, clay, silt, cohesion, NMC, sand, PI, phi and LL have strong interrelation in the correlation array.

XRD result revealed the presence of kaolinite clay minerals, even though kaolinite are the least of expansive clay minerals. Generally clay minerals have the tendency to add more plasticity to the soil, which is an important causes of foundation failure. It is anticipated that for engineering structures constructed on expansive soils, the size of cracks increase with time until it results to complete collapse. From field observation types of cracks visible in the buildings are vertical, diagonal and horizontal shear cracks caused by bending moments, horizontal and vertical tension cracks^19^.

Comparing the geotechnical parameters from the area of study with the Nigerian requirement for constructions, the area of study has substandard foundation materials and as so should be properly stabilized prior to construction activities on them.

## Recommendation

From the findings analyzed above, we propose that:

I. Thorough subsoil investigation for foundation stability should be carried out before the construction process.
II. Before erecting any structure, soils within the study area should be compacted on dry side of optimum moisture content, t0 achieve high shear strength and low compressibility.
III. Proper drainage system must be made available for surface runoff and to stop surface water infiltrations around the foundation of the building.
IV. Deep foundations have to be extended till a hard stratum is reached.
V. Future studies should look into further potential approaches to increase the stability of clay for construction.

## Data Availability

The datasets generated and/or analyzed during the current study can be made available upon reasonable request from the corresponding author because the author is still using the datasets for further studies**.**
